# Tumor versus Tumor Cell Targeting in Metal-Based Nanoparticles for Cancer Theranostics

**DOI:** 10.3390/ijms25105213

**Published:** 2024-05-10

**Authors:** Jesús David Urbano-Gámez, Cinzia Guzzi, Manuel Bernal, Juan Solivera, Iñigo Martínez-Zubiaurre, Carlos Caro, María Luisa García-Martín

**Affiliations:** 1 Biomedical Magnetic Resonance Laboratory—BMRL, Andalusian Public Foundation Progress and Health—FPS, 41092 Seville, Spain; j.urbano@ibima.eu (J.D.U.-G.); cinzia.guzzi@ibima.eu (C.G.); 2 Instituto de Investigación Biomédica de Málaga y Plataforma en Nanomedicina–IBIMA Plataforma BIONAND, C/Severo Ochoa, 35, 29590 Malaga, Spain; mbernal@uma.es; 3 Departamento de Biología Molecular y Bioquímica, Facultad de Ciencias, Universidad de Málaga, Andalucía Tech, 29071 Malaga, Spain; 4 Department of Neurosurgery, Reina Sofia University Hospital, 14004 Cordoba, Spain; juan.solivera.sspa@juntadeandalucia.es; 5 Department of Clinical Medicine, Faculty of Health Sciences, UiT The Arctic University of Norway, P.O. Box 6050, Langnes, 9037 Tromsö, Norway; inigo.martinez@uit.no; 6 Biomedical Research Networking Center in Bioengineering, Biomaterials & Nanomedicine (CIBER-BBN), 28029 Madrid, Spain

**Keywords:** metallic nanoparticles, cancer, biological barriers, tumor targeting in vivo, tumor cell targeting in vivo, theranostics

## Abstract

The application of metal-based nanoparticles (mNPs) in cancer therapy and diagnostics (theranostics) has been a hot research topic since the early days of nanotechnology, becoming even more relevant in recent years. However, the clinical translation of this technology has been notably poor, with one of the main reasons being a lack of understanding of the disease and conceptual errors in the design of mNPs. Strikingly, throughout the reported studies to date on in vivo experiments, the concepts of “tumor targeting” and “tumor cell targeting” are often intertwined, particularly in the context of active targeting. These misconceptions may lead to design flaws, resulting in failed theranostic strategies. In the context of mNPs, tumor targeting can be described as the process by which mNPs reach the tumor mass (as a tissue), while tumor cell targeting refers to the specific interaction of mNPs with tumor cells once they have reached the tumor tissue. In this review, we conduct a critical analysis of key challenges that must be addressed for the successful targeting of either tumor tissue or cancer cells within the tumor tissue. Additionally, we explore essential features necessary for the smart design of theranostic mNPs, where ‘smart design’ refers to the process involving advanced consideration of the physicochemical features of the mNPs, targeting motifs, and physiological barriers that must be overcome for successful tumor targeting and/or tumor cell targeting.

## 1. Introduction

Cancer encompasses a set of pathologies whose origins are not yet fully understood [1]. This disease continues to be one of the main causes of death worldwide, with a projected rising incidence in the coming years [2]. Its cellular basis involves genetic and/or epigenetic errors resulting in uncontrolled replicative capacity, as well as the development of mechanisms to evade contact inhibition and induce controlled cell death [3]. Malignant diseases can affect any part of the body, meaning that, essentially, all tissues are susceptible to developing cancer. Despite considerable pathological differences among various types of cancer, their effects on the body are similar [4]. Typically, except in the case of lymphatic and blood cancers (leukemias), mutated cells begin to multiply uncontrollably, forming a tissue mass (primary tumor) [5]. Its size would initially be limited to 1–2 mm due to restrictions in nutrient and oxygen supply [6]. However, tumor cells are capable of secreting factors that trigger the formation of new blood vessels from a preexisting vascular bed in a process called tumor angiogenesis, resulting in an unlimited supply of nutrients and oxygen and, consequently, unrestricted tumor growth [7]. If this progression were localized in a single organ or tissue, the public health problem caused by cancer would be much smaller. However, some cells from the primary tumor are capable of detaching, traveling through the body via the bloodstream or lymphatic system, settling in a distant location, extravasating into the perivascular connective matrix, and ultimately proliferating in the new tissue, forming metastatic nodules [8,9]. In fact, metastatic processes account for between 70% and 90% of cancer-related deaths [10,11].

A cancer diagnosis can be defined as the set of necessary tests to determine its presence, location, type, and stage. Initially, it predominantly relies on imaging tests, such as computed tomography (CT), magnetic resonance imaging (MRI), or ultrasound (US) [12], which provide valuable information on the characteristics of the tumor and its spread within the body. Subsequently, these imaging techniques are used to monitor the progression of the patients once diagnosed and/or treated [9,13]. Additionally, analytical tests of biofluids (blood, urine, feces, etc.) are used for diagnosis [14], and tissue tests (through biopsy or cytology) are devoted to both diagnosis and confirmation [15]. As for treatment, there is a plethora of therapeutic approaches to combating cancer, closely dependent on the type and stage of cancer as well as the individual characteristics of the patient [16]. In general terms, the main options are surgical resection of the tumor, chemotherapy, targeted therapy (druggable driver mutations), immunotherapy, and radiotherapy [17,18]. The order in which they are applied depends on the circumstances of the patients, but typically, two or more are used in tandem to treat the primary tumor and prevent or delay both tumor recurrence and metastatic processes [19].

In recent years, new approaches have come into play in the nanomedicine field. In particular, nanoparticles (NPs) for cancer therapy and diagnostics (theranostics) have been the subject of extensive research. The physical and chemical properties of a material, including its magnetic, mechanical, optical, and catalytic properties, are determined by both its structural and electronic characteristics. These properties can vary depending on the size of the material [20]. The concept of nanotechnology was first introduced by Prof. Richard Feynman (Nobel Prize in Physics in 1965) in 1959 during his famous lecture “There is plenty of room at the bottom” at the annual meeting of the American Physical Society at the California Institute of Technology (Caltech) [21,22]. Fifteen years later, Prof. Norio Taniguchi was the first scientist to use and define the term “nanotechnology” [23]. Nanotechnology is dedicated to observing, measuring, manipulating, controlling, and manufacturing matter (atomic and molecular structures) with at least one of its dimensions in the nanometer range (1 < 100 nm) [24]. By reducing a material to the nanoscale, there is an increase in the proportion of atoms located on the surface compared to those within the material [25]. This geometric phenomenon leads to a larger contact surface area of the material with the environment, thus resulting in greater chemical reactivity due to the increased number of unsaturated atoms with unsatisfied coordination, making them unstable and highly prone to combine [26]. Additionally, these surface atoms exhibit different local symmetry, known as different magnetic anisotropy [27]. Moreover, nanoscale size also involves other effects, such as increased Van der Waals attraction forces and quantum confinement in a specific direction [28]. Quantum confinement results in variations in the density of states at the Fermi level and consequently affects the intrinsic magnetic properties of materials [29]. This multidisciplinary research field has experienced rapid growth in recent decades due to its potential to revolutionize numerous areas, ranging from electronics and medicine to energy and material manufacturing [30]. Nanomedicine is an area within nanotechnology that provides new tools for the diagnosis, treatment, and prevention of diseases, with direct applications in medicine [31]. Leveraging the new properties of materials at the nanoscale and taking advantage of the fact that this size ‘per se’ falls within the dimensional range of biological entities with relevant functions (antibodies, membrane receptors, etc.), nanomedicine enables medical approaches from within the body at a cellular or molecular level in key areas such as diagnosis [32], gene therapy [33], tissue regeneration [34], drug delivery [35], and therapeutic approaches [36]. Indeed, a burgeoning field at the forefront of biomedical research has recently emerged, promoting a unique blend of theranostic approaches with single nanoscale entities, metal-based nanoparticles (mNPs), among the most applied in the field [37,38]. They can serve as contrast agents (CAs) in various imaging modalities, including MRI, CT, and photoacoustic imaging (PAI), facilitating early and precise cancer detection. Simultaneously, mNPs possess inherent physicochemical properties that make them ideal candidates for targeted drug delivery, hyperthermia-based cancer therapy, and photothermal therapy (PTT). Therefore, by leveraging the multifaceted nature of mNPs, they hold the potential to play a significant role in novel cancer treatment strategies that integrate imaging and therapy, ultimately leading to improved patient outcomes.

In this review, we will critically analyze some of the main challenges that must be overcome to achieve successful targeting (whether of tumor tissue or cancer cells within the tumor tissue), along with the crucial features required in the smart design of theranostic mNPs.

## 2. Tumor Targeting

The process of tumor targeting involves the capability of mNPs to reach and accumulate within the tumor tissue, irrespective of the administration route employed or the later cellular interactions. This phenomenon underscores the crucial ability of mNPs to localize/concentrate within the tumor microenvironment (TME), thereby enhancing their potential diagnostic and/or therapeutic utility. Consequently, there is a pressing need to comprehend and optimize this process. First of all, numerous barriers hinder the achievement of sufficient doses in solid tumors following the intravenous administration of mNPs in the majority of cases.

### 2.1. Physiological Barriers

#### 2.1.1. Blood

The first and possibly the most important barrier that mNPs encounter once they enter the bloodstream is the blood itself. Shortly after administration, specific proteins (opsonins) begin recognizing and tagging them for elimination through an immunological process called opsonization, part of the inflammatory response [39,40]. Among opsonins, the following stand out:

*Complement.* The complement is a system of plasma proteins (more than 30 different ones) that recognize foreign particles, bind to their surface, and trigger a cascade of signals, facilitating their recognition and elimination by immune system cells [41].

*Antibodies or immunoglobulins.* These are proteins produced by the immune system in response to specific antigens. There are two main types involved in opsonization: IgG and IgM. They specifically mark foreign particles for recognition by immune cells [42].

*Specific opsonizing proteins.* For example, C-reactive protein (CRP), produced in response to inflammatory processes, attaches to foreign particles for their recognition and elimination [43].

The multilayer of opsonins onto the mNP surface, known as the “protein corona”, forms rapidly (≈0.5 min) and continues for hours. This process provides mNPs with a new biological identity, altering their intrinsic physicochemical properties and thereby modulating their stability, circulation time, and cellular uptake/interactions, consequently affecting their functional role and biodistribution [44,45]. The protein corona has been previously shown to be influenced by the size/shape and chemical nature (surface properties, hydrodynamic diameter (HD), etc.) of the mNPs [46,47,48,49,50]. However, despite exhaustive documentation of the impact of mNP characteristics on the protein corona, it is not fully understood how the interaction of all these factors collectively influences its formation. Moreover, although many researchers in recent years have recognized the significant importance of the protein corona in determining the fate of mNPs once they are injected, it is still not entirely clear whether the composition of the protein corona, the relative amount of certain proteins, or other unknown factors determine it.

In summary, opsonization leads to both macrophages and neutrophils in the bloodstream phagocytizing foreign particles (such as mNPs) and removing them, drastically reducing their circulation times [51].

*Albumin.* Albumin belongs to the albumin superfamily, which comprises transport proteins such as α-fetoprotein, vitamin D-binding protein (Gc-globulin), and afamin (α-albumin). It is an aglycosylated, negatively charged protein composed of 585 amino acids, forming a single polypeptide chain with a molecular weight of 66.5 kDa, and it is the most abundant circulating protein found in plasma, representing half of the total protein content [52]. However, albumin is not typically considered an opsonin in the traditional sense; instead, it can function as either an opsonin or a dysopsonin [43].

#### 2.1.2. Specialized Tissues

**Figure 1:**
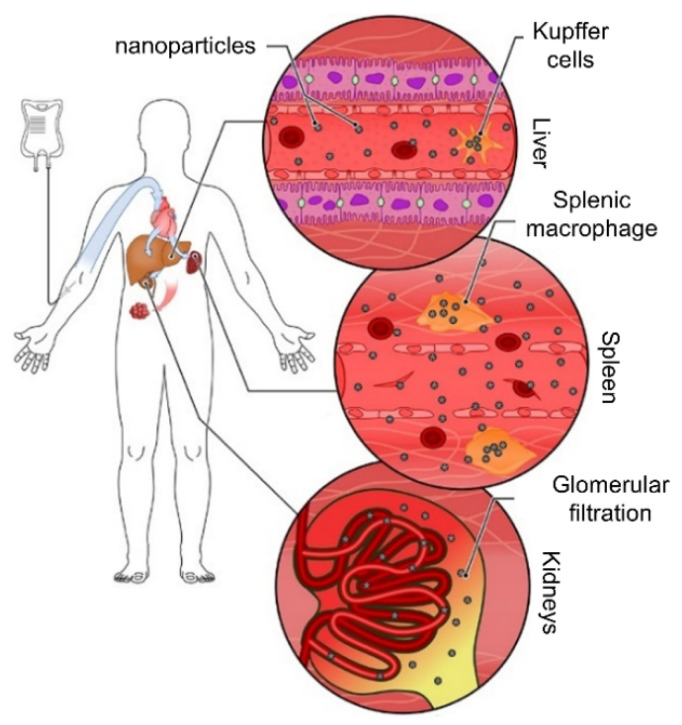
Systemic clearance of nanoparticles. Following intravenous injection, nanoparticles are distributed systemically through the bloodstream. They reach the liver and the spleen, where tissue-resident macrophages (called Kupffer cells in the liver) and endothelial cells (LSEC) sequester a large portion of the administered dose. Nanoparticles small enough to pass the glomerular filter (below ~5 nm) are excreted in the urine. The remaining nanoparticles have the opportunity to accumulate in tumor tissues [53]. Adapted. Copyright (2019) Frontiers.

The next barrier that mNPs face consists of specialized tissues, which, among other functions, purify the blood by removing foreign objects or defective cells. This process involves the kidney, albeit indirectly, as most mNPs are not directly filtered due to their size, and primarily the liver and spleen (detailed below), as depicted in Figure 1 [53].

*Liver.* This organ is primarily responsible for purifying foreign substances and particles in the blood. This process is carried out by the hepatic macrophages (Kupffer cells) and the endothelial cells in the hepatic sinusoids, also called liver sinusoidal endothelial cells (LSEC) [54]. Following the action of Kupffer cells, the main routes for mNP excretion are through feces (via biliary excretion) or urine (transforming mNPs into smaller and more water-soluble forms and subsequently releasing them into the bloodstream) [55].

*Spleen.* It plays a relevant role in eliminating foreign particles and removing damaged or aged blood cells. Splenic macrophages (sinusoidal), primarily present in the red pulp, are responsible for eliminating foreign particles from the blood [56]. In the particular case of iron oxide NPs (IONPs), they can be partially or completely recycled, incorporating the products into normal iron metabolism [57].

It is worth noting that blood macrophages, Kupffer cells, and splenic macrophages are part of the mononuclear phagocyte system (MPS) [58], emphasizing their prominent role in tumor targeting with mNPs. Originally, the concept of the MPS, also known as the reticuloendothelial system (RES), was introduced to classify macrophages found in various tissues (such as the gut, liver, lung, etc.), blood monocytes, and their precursors. This classification was based on multiple criteria, including morphology, function, origin, and kinetic properties [59].

#### 2.1.3. The Special Case of the Blood–Brain Barrier (BBB)

The blood–brain barrier (BBB) is a specialized and complex tissue structure that separates circulating blood from the central nervous system (CNS) [60]. Its main function is to protect the CNS by physically isolating brain tissue from the blood and, most importantly, by selectively regulating the exchange of substances between them. In this way, the BBB prevents the effects of potentially harmful or toxic substances, allows the passage of essential molecules (oxygen, glucose, and some amino acids), and maintains a stable environment (regulating ionic balance and homeostasis) for proper brain function [61]. From a physiological standpoint, the BBB consists of highly specialized endothelial cells that form the walls of the cerebral microvasculature. The junctions between these cells are tight and very stable, preventing the paracellular transport of molecules and cells into the brain [62]. Additionally, endothelial cells exhibit polarization between the luminal and abluminal regions, each with specific receptors to control the flow of molecules [63]. Furthermore, the endothelial cells of the BBB are surrounded by perivascular cells, similar to the microvasculature in the rest of the body, but with a significant difference: the ratio of perivascular cells to endothelial cells is up to a hundred times greater in the BBB [64]. Generally, perivascular cells provide stability to the microvasculature and also play an important role in regulating angiogenesis, inflammation, and blood flow [64]. Finally, the structure formed by endothelial and perivascular cells is surrounded by glial cells, mainly astrocytes, which contribute to BBB integrity and regulate its permeability [65]. The exchange of molecules across the BBB, not mediated by specific receptors, is essentially determined by two factors: their size and chemical nature [66]. Thus, lipophilic molecules with sizes smaller than 500 Da are capable of crossing the BBB practically without restrictions [63]. Conversely, certain molecules require selective transporters that limit/control their passage into the brain [67]. In fact, the BBB not only presents receptors that allow the entry of molecules into the brain but also, through the use of energy (adenosine triphosphate (ATP)), is capable of expelling molecules from this tissue, especially those that could be toxic, against a concentration gradient [68]. These are called “ATP-binding cassette” (ABC) transporters. Among them, one of the most widely studied is P-glycoprotein (P-gp), capable of reducing the intracerebral concentration of multiple drugs and thus limiting their therapeutic efficacy [69]. In addition to their role in substance transport, ABC transporters are also involved in various physiological processes, such as local metabolic regulation, immune response, and cellular homeostasis [70]. Indeed, overcoming the BBB remains one of the primary challenges in the development of mNPs for brain tumor theranostics.

#### 2.1.4. Tumor Itself

**Figure 2:**
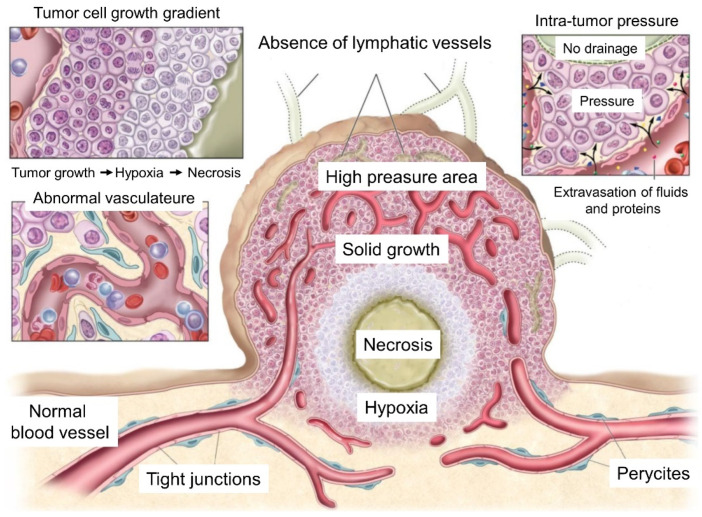
Physiological characteristics of tumor tissue and vasculatures that can facilitate or prevent cancer drug delivery [71]. Adapted. Copyright (2014) Ivyspring.

Besides the biological barriers discussed above, tumors themselves pose a formidable challenge in cancer theranostics with mNPs due to their complex microenvironment and heterogeneity, which create physical and biochemical barriers that impede mNP penetration and accumulation within the tissue. Indeed, understanding the role of tumors as barriers is crucial for developing strategies to enhance the efficacy of mNP targeting (Figure 2) [71].

On the one hand, the physical barriers mainly consist of the extracellular matrix (ECM), which is a dense and fibrotic network of proteins and carbohydrates providing structural support to tissues [72]. Furthermore, increased interstitial fluid pressure (IFP) poses an additional impediment to the efficient tumor uptake of mNPs. Increased IPF is due to the leakiness of neoangiogenic blood vessels, abnormalities (tortuosity, disorganization, leakiness, compression, etc.) in tumor lymphatic vessels, interstitial fibrosis, and matrix contraction mediated by stromal [73]. The inefficient and poorly developed vascular network in tumors represents, by itself, a significant physical barrier for NPs to reach tumor cells [74].

On the other hand, the biochemical barriers reside in the overexpression of certain proteins within the tumor. These proteins can either actively pump out mNPs due to variations in biochemical conditions (such as hypoxia) or degrade them through the enzymatic activity of secreted proteins. Moreover, the stability of mNPs can be modified by intrinsic features of the tumor promoted by its metabolism (such as pH imbalance), and the local immune response within the tumor tissue can lead to mNP clearance or altered biodistribution [75].

### 2.2. Passive Tumor Targeting

Solid tumors are characterized, among other features, by the abnormal development of blood and lymphatic vessels, which results in increased vascular permeability and reduced lymphatic drainage compared to normal tissues. For this reason, intravenously administered mNPs can more readily accumulate in the tumor tissue. This process is known as the Enhanced Permeability and Retention (EPR) effect and was first described by Prof. Maeda in 1986 [76]. In the field of nanotechnology, the EPR effect has been extensively studied for both diagnosis and therapy of solid tumors, aiming to improve efficacy and reduce side effects in healthy tissues [77]. In fact, it has been widely described as the most efficient mechanism for targeting mNPs in solid tumors [78,79,80,81]. However, it is also known that the EPR effect varies depending on the type of tumor, its developmental stage, and the heterogeneity of blood vessels [82].

Thus, the EPR effect remains the subject of considerable controversy. For instance, aberrant tumor vasculature may translate into poor tissue perfusion and inefficient dissemination of the therapeutic. Also, the increased interstitial pressure inside tumors is another physical barrier for passive tumor targeting. In 2014, Prof. Nichols commented that the attenuated impact of the EPR effect in the fight against cancer is likely the result of numerous factors, although none of them entirely invalidate it [83]. In this regard, some of us have recently demonstrated that the limitations observed in the EPR effect are even more pronounced in the context of brain tumors [84].

Nonetheless, in certain cases, the EPR effect can serve as a good basis for achieving significant accumulations of mNPs in the tumor through their intelligent design, capable of utilizing intrinsic tissue properties that lead to stability changes of mNPs, allowing them to remain retained for longer periods. An example is the work by Tseng et al. [85], in which they reported the development of a lactate-responsive mNP carrier as a therapeutic approach for treating non-small cell lung cancer (NSCLC) in a subcutaneous mouse model (H1975 cell line). Magnetized adeno-associated virus serotype 2 (AAV2) was loaded with lactate oxidase (LOX). Excessive lactate generated in the TME enters the permeable coating, where LOX oxidizes it to pyruvate inside the carrier. Additionally, the magnetic nature of the carrier facilitated their MRI tracking in vivo. Another similar strategy in which single oxygen (^1^O_2_) was generated as a cytotoxic agent in cancer PDT was proposed by Deng et al. [86]. They developed an MRI probe able to visualize the ^1^O_2_ generated by PDT. The MRI probe was synthesized by co-encapsulation of the photosensitizer compound chlorin e6 (Ce6) and Fe_3_O_4_ mNPs in mPEG-2000-Thioketal(TK)-C16 micelles, in which TK is highly sensitive only to ^1^O_2_ production and not to other ROS. Singlet oxygen production by light irradiation triggered the cleavage site of the TK; this reaction produced an increase in the hydrophobicity and an aggregation of the magnetic core of the mNPs, which enhanced the visual and specific detection of ^1^O_2_ generated by PDT using in vivo and in vitro models. In recent years, the nanotherapeutic properties of mNPs have been significantly enhanced by promoting their aggregation in the TME, as is the case of a very recent work in which Wei and co-authors synthesized mNPs functionalized with two ligands—3,4-dihydroxyhydrocinnamic acid (DHCA) and trimethylammonium dopamine (TMAD). The final mNPs were responsive to pH variations in the TME, leading to aggregation and an increase in diameter, thereby promoting their accumulation. This variation in diameter enhanced the MRI contrast capability, PTT efficiency, and PAI performance of the functionalized mNPs. The versatility of these mNPs could help better localize the primary tumor mass and improve its treatment [87].

### 2.3. Active Tumor Targeting

Active tumor targeting with mNPs refers to a procedure in which the accumulation of mNPs within the tumor is enhanced by various methods, such as applying external stimuli or modifying the NPs themselves. Different approaches employed for this purpose will be described in the following sections.

#### 2.3.1. Intratumoral Administration

Intratumoral administration, while providing direct delivery into the tumor mass, cannot be considered active in the conventional sense, as active targeting typically refers to strategies aimed at directing mNPs to tumors through the bloodstream. However, a new therapeutic approach called Convection Enhanced Delivery (CED) has emerged in the last few years, demonstrating promising therapeutic results after the intratumoral administration of formulation in brain tumors [88,89]. Therefore, intratumoral administration, although it does not strictly conform to the concept of active tumor targeting, should not be disregarded as a viable alternative for tumor therapy, especially in the context of brain tumors [90].

#### 2.3.2. External Stimuli

**Figure 3:**
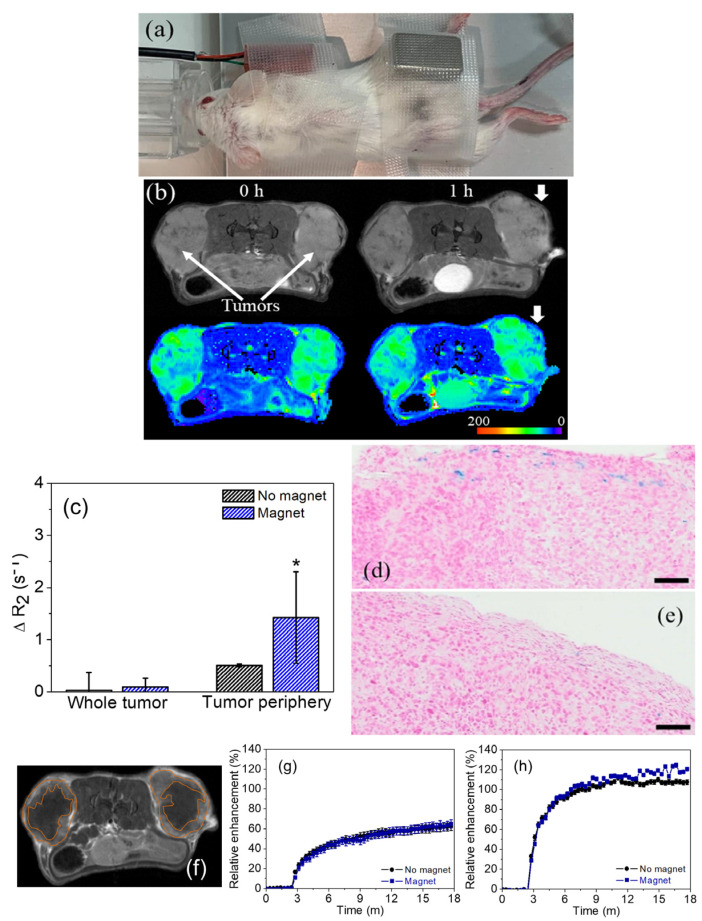
(**a**) Magnetic tumor targeting as a proof-of-concept experiment. (**b**) Representative T_2_-weighted MR images (**top**) and parametric T_2_ maps (**bottom**) at 0 and 1 h after NP injection. Thick arrows indicate the main zones of magnetically-guided accumulation of the NPs (tumor periphery). (**c**) ΔR_2_ (s^−1^) at the whole tumor or tumor periphery at 1 h post-intravenous administration of the NPs, calculated from the quantitative T_2_ map analysis. Differences were considered statistically significant at *p* < 0.01 (*), compared to 0 h. PB staining of histological sections of the tumors, 1 h after NP administration, and with (**d**) and without (**e**) magnetic targeting. Bar lengths: 100 μm. Representative T_1_-weighted MR image (**f**) and short-term MRI characterization of the distribution of Gadovist^®^ in the whole tumor (**g**) and tumor periphery (**h**), drawn with orange lines, after its intravenous administration. The blue lines in g and h refer exclusively to the tumors where the NPs were targeted magnetically before. Differences in the values (%) could be attributed to minor vascularization differences in the area selected manually, which could contain a necrotic zone where Gadovist^®^ may show a slower enhancement curve [93]. Adapted. Copyright (2023) Elsevier.

The aim of this active targeting approach is to direct the mNPs to the solid tumor based on their responsiveness to external stimuli. This process increases the amount of mNPs in the tissue while potentially decreasing side effects. Undoubtedly, the targeting of magnetic materials by applying external fields is the most widely studied strategy within this topic. Nevertheless, a key aspect must be considered in this context: if the magnetic interaction between mNPs is strong enough, it could result in thrombus formation once they are in the bloodstream, with consequent adverse effects on the patient, including potential fatality. Some of the more recent works concerning external stimuli-based active targeting will be described next, but readers are encouraged to consult other works where the topic has been extensively reviewed [91,92]. An enhancement in tumor targeting following the exposure of mNPs to a magnetic field compared to passive targeting was reported recently in a heterotopic model (Figure 3) [93]. While successful active targeting was achieved, the study demonstrated that the accumulation of mNPs mainly occurred at the tumor surface (close to the magnet place), showcasing the potential limitations of this approach in reaching the optimal (homogeneous) distribution of mNPs within the tumor.

In addition, an improvement in magnetic tumor targeting was observed in orthotopic models compared to heterotopic models [94], evidencing that tumor targeting depends on the tumor type, as previously mentioned in this review. However, magnetic tumor targeting is just the tip of the iceberg regarding accumulation based on external stimuli. For instance, an intriguing study described mNPs capable of aggregating on demand, triggered by light irradiation through a tetrazole/alkene cycloaddition reaction [95]. This process resulted in greater retention within the solid tumor.

#### 2.3.3. Transcytosis through Vascular Endothelial Cells

In the context of mNPs and tumor targeting, transcytosis refers to the transcellular transport of mNPs through the endothelial cells lining the tumor capillaries, involving their internalization on the luminal side and their subsequent release on the abluminal side, thereby reaching the tumor interstitium. Different mechanisms facilitate transcytosis through the vascular endothelial cells, such as receptor-mediated or caveolae-mediated pathways, with the former being the most commonly used in mNPs for active tumor targeting. Receptor-mediated transcytosis involves the recognition of receptors on the luminal surface of endothelial cells, triggering receptor-mediated endocytosis, followed by the transport of the vesicles containing the mNPs across the cytoplasm and, finally, exocytosis on the abluminal side [96]. Among the receptors used for active tumor targeting via receptor-mediated transcytosis, integrins and the transferrin receptor stand out.

*Integrins.* Integrins are heterodimeric receptors composed of an alpha and a beta subunit [97]. They are present on the cellular surface, mediating cell interactions with the structural proteins of the ECM [98]. Integrins are often overexpressed in the endothelial cells of tumor vasculature, making them a potential target for active tumor-targeting strategies [99]. Among the ligands of integrins are ECM proteins like collagens, laminins, and fibronectins, many of which share an integrin-binding motif formed by arginine-glycine-aspartate (RGD) [98]. Thus, RGD is the functionalization motif most commonly used to target tumor endothelial integrins [100,101]. However, while numerous studies discuss the use of RGD motifs to promote active tumor targeting via endothelial transcytosis in polymeric nanoparticles and nanoliposomes, only a few studies report this approach in metallic nanoparticles. For instance, gold nanoparticles functionalized with RGD exhibited successful lung tumor targeting upon intratracheal administration [102]. Additionally, another interesting application is the functionalization of iron oxide nanoparticles with RGD to improve MRI-based tumor diagnosis [103,104].

Of note, RGD is not only overexpressed in tumor endothelial cells but also in several tumor cells, thus being reported in numerous studies as a motif for tumor cell targeting, as will be discussed later.

*Transferrin receptor.* Transferrin is an iron-transporter glycoprotein that binds to the transferrin receptor, which mediates its endocytosis to transport iron molecules into the cells [105]. Amidst tumor targeting, transferrin has gained significant popularity, particularly in the exploration of its application for brain malignancies, due to its ability to penetrate the BBB via receptor-mediated transport [106]. In this regard, ultrasmall IONPs functionalized with lactoferrin (a member of the transferrin family) were shown to accumulate within C6 rat gliomas [107]. Moreover, glioma targeting with mNPs through transferrin functionalization has been nicely reported and analyzed previously [108].

#### 2.3.4. Other Active Targeting Strategies

Other mechanisms to enhance the accumulation of mNPs within tumors include specific interactions with cellular or non-cellular components of the TME that are abundantly and exclusively expressed in tumor tissues, such as fibroblasts (CAFs) or tumor-associated macrophages (TAMs) [109]. In the context of interaction with cellular components, an exciting approach was proposed by Lee et al. [110]. Their strategy relies on the recruitment capacity of immune cells—immature myeloid cells (iMCs), neutrophils, and TAMs—by tumor cells. The authors developed a click reaction-assisted immune cell targeting (CRAIT) strategy to enhance the delivery of the drug-loaded NPs into the avascular regions of the tumor. The CRAIT strategy was applied to an orthotopic 4T1 tumor mouse model. CD11d antibodies were functionalized with trans-cyclooctene (TOC) to enable biorthogonal click reactions with mesoporous silica mNPs functionalized with tetrazines (MSN-Tz). These modified antibodies were administered via tail vein injection 24 h before the administration of NPs. Subsequently, MSN-Tz was injected following the same route and was able to specifically recognize CD11+ cells. This strategy significantly reduced the tumor burden after treatment. On the other hand, non-cellular elements from the ECM have also been considered as putative targets for developing an anti-cancer strategy. Thus, Murty and co-authors developed mNPs in which the most external layer of the coating was attached to a collagenase protein. The final goal of these mNPs was to enhance their accumulation in the TME because the integrity of the dense surrounding stroma was compromised by the collagenase activity of the functionalized mNPs [111].

### 2.4. Direct Effect on Physiological Barriers

As previously described, the MPS is the first barrier that mNPs face upon intravenous administration. An attractive approach to overcoming this barrier is its saturation. Weaver et al. conducted a compelling study on the potential MPS saturation using unfunctionalized yet water-stable mNPs [112]. Through repeated mNPs dosing (once weekly for 8 weeks), the authors observed no saturation of either Kupffer cells or splenic macrophages. Therefore, this strategy of repeated administration failed to surmount the MPS and should not be considered a promising approach to enhance tumor targeting. Moreover, adjusting the quantity of administered mNPs until reaching a predetermined threshold increases tumor targeting by saturating the MPS. In this context, a general threshold of 1 trillion mNPs has been recently identified as the quantity that surpasses the capacity of the liver [113]. These data open new avenues in the development of theranostic mNPs, but caution must be exercised as the toxicity of this large quantity of mNPs is not yet clear. Overall, while both strategies are highly interesting, further in-depth studies are necessary to comprehensively assess their efficacy, potential side effects, and long-term implications.

An interesting alternative approach for active tumor targeting involves the application of external forces or elements that affect the intrinsic features of the (intra)tumoral barriers, resulting in their disruption or breakdown. On the one hand, focused ultrasound (FUS) stands as a promising strategy to enhance mNPs delivery either in different tumor entities for improving perfusion, extravasation, and ECM transport of drugs [114] or into the brain by expanding or disrupting the tight junctions of the BBB. Nevertheless, the mechanisms through which nanoparticles extravasate through the opened gaps in the BBB are not fully understood [115]. Although there is limited literature on the application of FUS to enhance tumor targeting with mNPs, some studies have reported surprising accumulation percentages relative to the injected dose. For instance, gold–copper nanoclusters demonstrated accumulation percentages of ≈1.7% [116], and gold NPs with various sizes exhibited an accumulation of <0.5% [117]. However, FUS presents a strong limitation since the temporary disruption/permeabilization of the BBB may lead to unintended consequences, such as an increased risk of neurotoxicity due to the influx of potentially harmful substances. On the other hand, there is evidence that both hyperthermia and radiotherapy (when given at low doses) can enhance the EPR effects by modifying the tumor vascular structure, IFP, and tumor-associated macrophages (TAMs), as recently reviewed [118,119], thus breaking the tumor barrier. Nevertheless, a high percentage of the reported works described this pre-conditioning of the solid tumor for intravenous administration of liposomes, and, therefore, these results must be carefully considered due to the limitations of these nanostructures, which will be discussed later in this work. Concerning mNPs, few works explore this pathway, which could be an important and unexplored niche. This might open new avenues to improve tumor targeting with mNPs.

### 2.5. Concluding Remarks on Tumor Targeting

A summary of the main works cited in this review is presented in Table 1, outlining the nanoparticle composition, their physicochemical characterization, functionalization, targeting strategy, and targeting efficacy.

Several observations can be drawn from this table. First, it becomes evident that a comprehensive characterization of the nanosystem is not always provided, and parameters that could potentially determine its fate in vivo, such as size or charge, are often overlooked. Second, a qualitative rather than quantitative evaluation of tumor targeting efficacy is provided in most studies, making it difficult to compare across studies and objectively assess the actual targeting effectiveness of the nanosystem. Finally, based on the information provided for each of the discussed mNPs, it can be concluded that the ideal structure would encompass the following features: spherical shape and size below 30 nm (as determined by TEM), HD below 50 nm, active over passive targeting strategy, functionalization with targeting motifs rather than other active targeting mechanisms, and cores trackable by most common diagnostic imaging modalities, namely MRI or CT.

### 2.6. Designing NPs for Tumor Targeting

As underscored throughout this review, overcoming, or bypassing the MPS is imperative to achieving successful tumor targeting. In addition to the strategies discussed in the previous section, another, and perhaps the most widely used, mechanism to evade MPS involves functionalizing the surface of mNPs with certain molecules/compounds. This process has been extensively studied in the last few years; thus, clear and well-structured information can be found elsewhere [120,121,122,123,124]. Hence, only a brief description of the main coating ligands will be provided in this review:

*Surfactants.* They can be described as chemical compounds with an amphiphilic molecular structure, meaning they have a hydrophilic and a hydrophobic part (of a short chain). They adsorb to the surface of mNPs, constituting a layer that enhances colloidal stability (modifying the surface charge and solubility). Additionally, they can introduce functional groups on the surface of mNPs for further conjugations [125]. However, they have often shown some toxicity, limiting their biomedical applications [126].

*Lipids.* They have a structure similar to surfactants but with a longer chain. They can form surface layers onto the surface of mNPs or lipid bilayers in which NPs are encapsulated, called liposomes (or nanoliposomes) and similar to those in biological membranes [127]. Of note and remarkably, liposomes are undoubtedly the most approved nanoparticles by the U.S. Food and Drug Administration (FDA) and European Medicines Agency (EMA) for drug delivery in the clinical field (not only cancer) and, consequently, the most broadly used [128]. However, despite their distinguished improvement regarding drug delivery to fight cancer, challenges remain in regard to their stability, drug loading capacity, controlled release pharmacokinetics, and targeting efficiency [129,130].

*Polymers.* These organic compounds are the most extensively used for functionalizing metallic NPs, as their physicochemical properties allow improvements in various aspects, such as colloidal stability and the introduction of functional groups for subsequent chemical modifications. Additionally, there are biodegradable polymers and stimuli-responsive polymers (responsive to changes in pH, temperature, or ionic concentration) that enable the controlled release of drugs associated with the mNPs [131]. The most commonly employed polymers include chitosan, dextran, polyethyleneimine (PEI), and polyethylene glycol (PEG). Regarding tumor targeting, PEG stands out among polymers mainly due to its remarkable stealth properties (by preventing opsonization) [132] and the possibility of further functionalization [133]. The reduction in opsonization not only decreases the potential inflammatory response derived from mNP injection but also increases its circulation time in the bloodstream [134]. However, it is worth highlighting that the surface conformation of PEG strongly determines its stealth properties [135], an aspect that is seldom taken into account. Moreover, recent data from our laboratory revealed that even with the presence of this polymer, a protein corona forms with over 1000 different proteins (unpublished data), stressing the need for developing/finding other polymers with better stealth properties and gaining deeper knowledge into how the protein corona is formed and determines the in vivo fate of mNPs.

In summary, the smart design of mNPs for tumor targeting, with a focus on intravenous injection as the administration route, must prioritize a safe dosage capable of either saturating the MPS or possessing stealth properties sufficient to minimize their recognition by the MPS. Additionally, the mNPs might/should incorporate elements for active tumor targeting, achieved either through external forces, tumor-specific conditions that promote their retention, or directly through certain motifs strongly demanded by the tumor tissue, such as a nutrient. In the case of the BBB, these mNPs should also be functionalized with a certain compound/motif that specifically recognizes a transporter of this physiological structure, facilitating their transport through it. Finally, once the mNPs have reached the tumor, they must avoid the ABC transporters to prevent their return to the bloodstream.

## 3. Tumor Cell Targeting

Cell targeting, as part of the development of theranostic mNPs and their application in vivo, refers to the proficiency of functionalized mNPs with targeting motifs to specifically interact with cellular components, regardless of whether tumor targeting is favored. However, it is essential to note that cancer cells reside within the tumor tissue, and, consequently, without effective tumor targeting, cell targeting becomes futile.

### 3.1. Targeting Motifs

**Figure 4:**
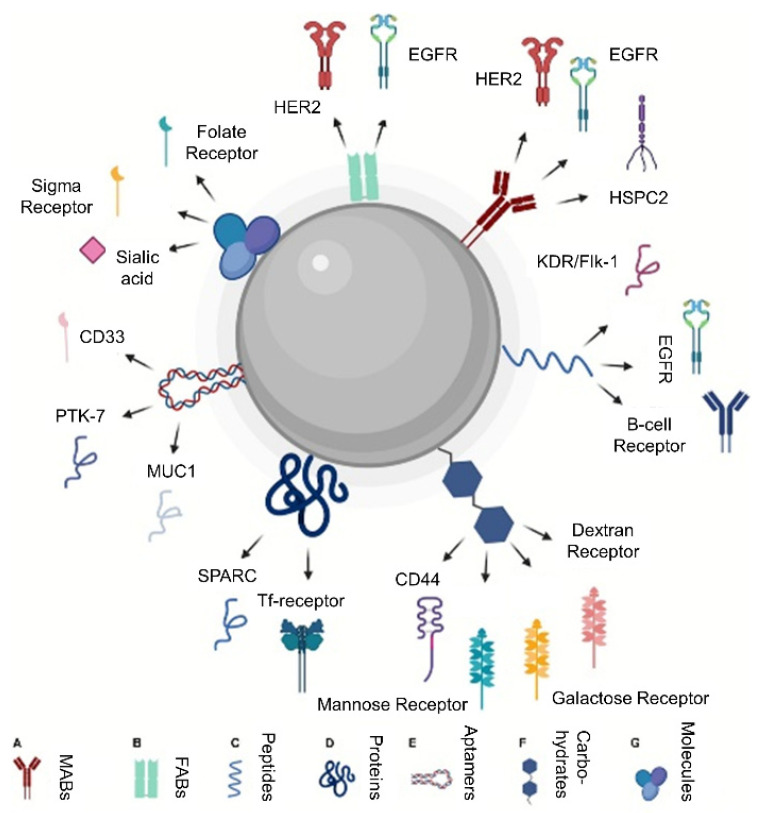
Design of nanoparticles for active uptake (created with BioRender.com). (A) Monoclonal antibodies, (B) fabs, (C) small peptides, (D) natural proteins, (E) aptamers, (F) carbohydrates, and (G) small molecules [124]. Adapted. Copyright (2020) Frontiers.

Numerous strategies exist for mNP functionalization, primarily leveraging adhesion molecules with overexpression in tumor cells of the primary mass. These approaches typically utilize crosslinkers/adapters between the ligand and mNP core or, less commonly, involve direct ligand binding to the mNP surface (Figure 4) [124]. A wide array of molecules has been employed for mNP functionalization, a selection of which will be discussed in this section.

#### 3.1.1. Antibodies

Antibodies are heterodimeric glycoproteins composed of four peptide chains (monomers), two heavy chains, and two light chains, the last constituting the responsible elements of antigen recognition [136]. Nowadays, immunotherapy has acquired high relevance in the development of tumor treatments [137]. Therefore, the use of antibodies (or their fragments) as motifs for targeting mNPs is a hot topic [138]. There are different strategies for the functionalization of mNPs with antibodies, as nicely reviewed elsewhere [139]. Thus, in this review, we will focus on cancer cell targeting after intravenous administration in cancer models:

*Direct antibody binding by carbodiimide chemistry.* This approach is based on the covalent binding of amine groups, which are exposed (lysines and at the N-terminal residue of light and heavy chains) to either aldehydes or carboxylic acid groups by applying a crosslinker (such as carbodiimide) [136,140]. Despite being the most frequently used strategy in mNP functionalization with antibodies, it can disrupt or reduce the affinity of the antibody to its antigen. This is because many of the reactive amine groups are localized in the light chains or close to them in the heavy chains of the antibody, and this interaction can alter the epitope-binding site, interfering with antigen recognition [141,142].

Regardless of this potential limitation, this approach was conducted for the targeting of breast cancer cells with gold NPs and IONPs functionalized with an anti-HER2 antibody [143,144,145]. Modest or even no differences were observed in the tumor accumulation comparing functionalized and non-functionalized mNPs, with the main results showing a prolonged residence time in the tumor. These results underscore the ineffectiveness of targeting tumor cells without first considering tumor targeting, as discussed above. Another study described the use of the C225 antibody to target the epidermal growth factor receptor (EGFR) in a mouse tumor model overexpressing this receptor (A549). C225 was linked to N-succinimidyl S-acetyl-thioacetate (SATA) by carbodiimide chemistry for the subsequent functionalization of PEGylated gold NPs. This process increased mNP accumulation within the tumor by more than twofold compared to non-functionalized mNPs, but it also increased mNP accumulation in other tissues [146]. This result highlighted the need for careful consideration when targeting EGFR, given its widespread expression across nearly all tissues.

Interestingly, the same approach, using carbodiimide crosslinkers, could be applied to bind mNPs to carboxylic groups. In the antibody, carboxylic groups are localized in the heavy chains inside the constant region (Fc) [136]. Although modifications to the Fc region could impact antigen-antibody recognition, this strategy could increase reproducibility and specificity in the antibody-NP orientation and reduce the risk of immunospecificity and immunoaffinity loss compared to binding through amine groups [147]. However, to our knowledge, there is no evidence in the literature supporting this approach.

*Direct antibody binding by sulfhydryl groups.* This strategy implies the covalent binding of the antibody through sulfhydryl groups (thiols), which are present in cysteine residues [138]. The sulfhydryl groups are responsible for the quaternary structure of the antibody, forming disulfide bonds [148]. However, once they are chemically reduced, they break to form thiolates, which are highly nucleophilic and can bind directly to mNPs, especially but not limited to gold and silver NPs, or through crosslinkers. Although maleimides are the most frequently used crosslinkers [140], haloacetyls are also used in antibody labeling [149]. Nevertheless, this approach exhibits some limitations, such as the breakage of disulfide bonds, which can affect antigen-antibody recognition [150], or the low stability in blood plasma of the maleimide-based conjugates [151]. In fact, this might be the reason for the lack of studies reporting the use of this strategy in targeting experiments conducted in vivo.

*Direct antibody binding by click chemistry.* This approach is based on the azide-alkyne Huisgen cycloaddition modified with catalytic agents (e.g., Cu and Ag) [152], strain-promoted cycloaddition [153], inverse electron demand Diels-Alder reactions [154], Staudinger ligations [155], and thiol-ene reactions [156]. It promotes highly efficient and regioselective reactions.

This chemical approach for the functionalization of mNPs enabled the detection of very small lesions at primary and lymphatic metastatic sites in an orthotopic model of gastric tumors using upconversion (NaGdF_4_:Yb,Er@NaGdF_4_) NPs [157]. Nevertheless, the lack of control mNPs in the in vivo studies limits the interpretation of their findings.

*Indirect antibody binding by avidin-biotin interaction.* Biotin forms a high-affinity complex with the avidin molecule or analogs. Hence, gold nanorods were functionalized with neutravidin for interaction with biotinylated anti-CD11b antibodies. These mNPs bound to neutrophils, which were carried to the TME after photosensitization in mouse models [158]. However, it is worth noting that this targeting method presents the limitation of being dependent on the previous photosensitization of the tumor area, thus posing doubts on whether the improvement in tumor targeting can be attributed to the functionalization of mNPs or the disruption of the tumor barrier by radiation.

#### 3.1.2. Ligands Binding Integrins

Besides their role in active tumor targeting, as discussed above, integrins are also used in the context of tumor cell targeting since the expression of certain integrins and their ligands is deregulated in some tumor cells, making them a potential target for tumor therapy [159].

In this sense, an iron-based metal-organic framework (MOF) bound to buthionine sulfoxide amine (BSO) and oxaliplatin (OXA) and covered by a lipid bilayer was functionalized with the RGD motif to improve tumor cell targeting. The effectiveness of the RGD targeting was evaluated using a fluorescent dye attached to the MOF, resulting in an increased accumulation of mNPs in the tumor tissue (heterotopic 4T1 tumor-bearing mice) compared to non-functionalized MOFs [160]. While fluorescence imaging demonstrates tumor accumulation of the dye within the tumor, the lack of assessment of concurrent iron and/or platinum accumulation in the tumor by MRI or ICP-MS raises doubts on whether the entire MOF reached the tumor or just free dye released from the MOF to the bloodstream. Additionally, palladium nanoparticles were functionalized with RGD, demonstrating significant targeting of breast cancer cells in vitro along with clear tumor accumulation in vivo, as measured by PA [161]. Despite these promising results, it is important to note that tumor cell targeting was assumed rather than confirmed, as the tumor tissue was not evaluated from a cellular point of view.

Moreover, it is worth mentioning that targeting integrins faces the challenge of a restricted battery of integrins and the redundancy in ligand recognition, which can be considered limiting factors, especially when compared to, for example, antibodies [162].

Because the focus of this review is not to analyze each specific functionalization but rather to focus on targeting concepts, readers can find all relevant information about mNPs functionalized with RGD in a previous work, bearing in mind that the authors did not distinguish between tumor targeting and tumor cell targeting [163].

#### 3.1.3. Glucose

The increased energetic metabolism is a hallmark of cancer and, therefore, becomes an important target in the design of diagnostic tools against cancer. The paradigm of this fact is the use of [18^F^]fluorodeoxyglucose in Positron Emission Tomography (PET) for medical imaging in cancer diagnosis [164].

However, functionalization with glucose has rarely been applied in nanomedicine for either tumor diagnosis or treatment. For instance, gold NPs functionalized with different amino-2-deoxy-D-glucose derivatives were able to accumulate in solid tumors due to the unique characteristics of tumor vasculatures. This accumulation contrasts with the one observed for [18^F^]fluorodeoxyglucose, preventing false positives, as this later molecule tends to accumulate in inflammatory processes [165]. In another study, iron oxide NPs were functionalized with Ce6 and glucose [166], providing effective PDT in heterotopic models of lung cancer. However, the tracking of mNPs was only conducted by fluorescence techniques, which consequently impedes the evaluation of whether Ce6 reached the tumor linked to the IONPs or as a free molecule. Furthermore, in another heterotopic model of lung cancer, the accumulated amount of iron in different tissues was evaluated over time after the intravenous administration of IONPs functionalized with glucose [167], showing a significant increase in the case of glucose-functionalized mNPs.

#### 3.1.4. Transferrin

Similar to ligand-binding integrins, transferrin is also utilized in tumor cell targeting due to its deregulated expression in certain tumor cells. In this sense, the transferrin receptor is overexpressed in some types of tumors, including colon [168], pancreatic [169], and lung cancers [170], making them potential candidates for tumor cell targeting with mNPs functionalized with transferrin.

However, despite the encouraging outcomes reported in some studies, such as the one above, there is controversy surrounding the use of transferrin as a targeting element in mNP functionalization, as it has been shown that its targeting capabilities are strongly affected by the formation of a protein corona [171].

#### 3.1.5. Aptamers

Aptamers are single-chain DNA or RNA oligonucleotides that acquire unique three-dimensional structures, conferring affinity to bind other molecules through ionic interactions, van der Waals forces, hydrogen bonds, or hydrophobic interactions [172], and even being able to differentiate enantiomers [173]. In contrast to immunoglobulins, they are more versatile and exhibit low or even non-immunogenic properties [174,175].

Aptamers continue to garner interest in nanomedicine as versatile molecules for mNP functionalization, benefiting from their unique features, as comprehensively examined earlier [176]. Surprisingly, out of more than 200 works, fewer than 10 are related to mNPs.

Despite this limited application to mNPs, some promising achievements have been made. For instance, gold NPs functionalized with an aptamer were capable of conducting targeted delivery of functional proteins, resulting in a delay of tumor growth in a heterotopic model of hepatic cancer [177]. Furthermore, a similar aptamer-nanotheranostic structure promoted a delayed response through the delivery of curcumin in a heterotopic model of colon cancer [178]. Another encouraging study reported doxorubicin-loaded hollow gold NPs coated with mesenchymal stem cell membranes to evade the MPS. They were later functionalized with an aptamer that recognizes mucin-1, resulting in successful tracking by CT and fluorescent imaging, as well as the treatment of tumors in a heterotopic model of breast cancer. The mNPs accumulated 24 h after administration, as confirmed by both CT and fluorescent imaging, suggesting that the mNPs maintained their stability until reaching the tumor tissue [179].

Regarding IONPs, when functionalized with prostate-specific membrane antigen (PSMA)-aptamers, they exhibited specific binding to target cancer cells compared to other nonspecific aptamers, as demonstrated by MRI in a heterotopic model of prostate cancer [180]. Additionally, these aptamer-functionalized IONPs were able to delay primary tumor growth through doxorubicin delivery. In another study, both IONPs and gold NPs were encapsulated inside mesoporous silica NPs along with elesclomol. This nanoformulation was functionalized with an aptamer that recognizes the epithelial cell adhesion molecule for multimodal imaging and therapy of a PC-3 tumor model. Although these mNPs had less effect on the tumor compared to free elesclomol, they showed reduced accumulation in healthy tissues and were associated with reduced side effects [181].

Silver NPs have also been functionalized with aptamers. This construct exhibited a high degree of accumulation in an orthotopic model of glioblastoma multiforme (GBM), later acting as a radiosensitizer that resulted in an outstanding therapeutic approach [182].

It is worth mentioning that aptamer-functionalized gold-coated magnetic NPs reached a therapeutic dose in a heterotopic model of Ehrlich’s carcinoma, successfully controlling tumor growth [183]. Regrettably, the absence of a control aptamer limits the interpretation of their findings, making it challenging to determine if the observed effects were due to direct interaction with tumor cells or simply to the accumulation of mNPs via EPR.

#### 3.1.6. Folate

Folic acid plays a crucial role in 1C metabolism, which is vital for the cell cycle [184]. The folate receptor is overexpressed in various tumors [185], making folic acid particularly relevant in both cancer diagnosis with radioisotopes [186] and chemotherapy [187].

Regarding active tumor targeting of mNPs functionalized with this molecule, there are a myriad of different nanoformulations. A recent study described that functionalized IONPs, carrying different chemotherapeutic compounds, were able to reduce tumor growth and alter the immune response within tumor tissue [188]. Furthermore, mesoporous silica-gold mNPs conjugated to folate were favorably applied for the targeted delivery of doxorubicin, promoting a delay in tumor growth [189]. In addition, iridium oxide mNPs combined with Ce6 and functionalized with folate were used for active tumor targeting of osteosarcoma cells in a heterotopic mouse model, resulting in tumor volume reduction [190]. Other examples include the targeting with folate of isoreticular metal-organic framework (IRMOF-3)-based mNPs carrying disulfiram [191] and iron oxide-bismuth sulfide mNPs [192]. These NPs were able to reduce tumor growth in a xenograft tumor model of Cal 27 adenosquamous carcinoma and a 4T1 breast cancer model, respectively.

Unfortunately, no in vivo imaging techniques were used to track the fate of the mNPs, as is also the case for many of the studies discussed throughout this review. Therefore, neither tumor nor tumor cell targeting could be directly confirmed in vivo. Additionally, tumor volume was often determined using a caliper, which is known to have inherent limitations in accuracy.

#### 3.1.7. Hyaluronic Acid

Hyaluronic acid is a glycosaminoglycan composed of β-1,4-D-glucuronic acid and β-1,3-N-acetylglucosamine units, primarily involved in structural functions in tissues [193]. However, recent studies have proposed many other functions, such as mediating and regulating cellular functions [194]. Hyaluronic acid is recognized by a plethora of different receptors, with particular interest in Cluster of Differentiation 44 (CD44), although the majority of them are overexpressed in tumor tissues [195]. In fact, CD44 expression correlates with increased tumor aggressiveness and metastasis generation [196].

Based on this rationale, the functionalization of mNPs with hyaluronic acid is a promising approach for targeting cancer cells upon intravenous administration and has thus been widely studied [197].

Thus, doxorubicin-loaded gold nanorods were functionalized with hyaluronic acid fragments to actively target breast adenocarcinoma cells in a heterotopic mouse model [198]. Similarly, gallium-indium mNPs carrying benzoporphyrin derivatives (a photosensitizer for PDT) were functionalized with hyaluronic acid. These mNPs were capable of completely eliminating tumors in a heterotopic model of pancreatic cancer after near-infrared irradiation [199]. However, while this therapeutic approach presumably targets only the irradiated area, evaluating healthy tissue remains critical to ensuring treatment specificity. Another study demonstrated the ability of zinc oxide mNPs coated with glucose oxidase and artesunate and then functionalized with hyaluronic acid to reduce tumor growth with negligible side effects [200]. Also, improved tumor targeting, as measured by near-infrared (NIR) PAI in the second biological window, and tumor eradication were obtained by functionalizing gold-coated manganese porphyrin mNPs with hyaluronic acid [201]. Nevertheless, the release of porphyrins prior to tumor accumulation was not evaluated, which could lead to an overestimation of tumor targeting. Finally, in a particularly intriguing study, gold nanorods were conjugated with 5-aminolevulinic acid (photosensitizer for PDT) and the dye Cy7.5 for image tracking. These nanorods were further functionalized with both hyaluronic acid and anti-HER2 antibodies, leading to a notable increase in tumor targeting, reaching 13% in comparison to the injected dose [202].

#### 3.1.8. Concluding Remarks on Tumor Cell Targeting

Overall, and without intending to be redundant or overly critical, the following observation can be gleaned from the majority of studies focusing on tumor cell targeting with mNPs. In most cases, the efficacy of the tumor cell targeting approach was anticipated rather than conclusively established, as it is predominantly evaluated in vitro in cell culture experiments or indirectly through the assessment of the antitumoral activity of the functionalized mNPs, lacking experiments that definitively validate if this effect is actually due to interaction of the mNPs with tumor cells promoted by their functionalization with cell-targeting motifs (for instance, electron microscopy imaging showing internalization of mNPs within tumor cells). On the other hand, it is imperative to bear in mind that for tumor cell targeting to occur, mNPs must first be able to reach the tumor interstitium, either passively through pores opening in the leaky angiogenic vasculature or actively through the various mechanisms described earlier. However, in most studies where particles are designed for “active targeting”, this actually refers to cellular targeting, overlooking the necessary prior step of tumor targeting.

### 3.2. Designing mNPs for Tumor Cell Targeting

As just mentioned, tumor targeting is imperative before cell targeting; therefore, mNP design should be aligned with this rationale. Then, an appropriate target must be identified to minimize side effects in other tissues as much as possible. Therefore, a previous comprehensive study to identify the motifs specifically overexpressed in the targeted tumor is mandatory. Strikingly, most studies overlook this characterization, as the overexpression of certain receptors is often assumed. However, it is worth noting that the high mutation rate of cancer cells could lead to changes in the expression of targeting motifs over generations [203]. Additionally, in the context of therapy, a crucial parameter that must be taken into consideration is the retention rate. Thus, if a mNP is able to target the tumor specifically but has a low retention rate (low affinity), there may not be enough time for the compound to exert cytotoxic effects on tumor cells. Moreover, the concept of the ‘binding site barrier’ must be kept in mind when designing mNPs for tumor cell targeting, as the targeting motifs may have critical effects on biodistribution once mNPs are intravenously administered. The binding site barrier refers to the high affinity of the binding molecules to their ligands, thus limiting the infiltration capacity of the mNPs into the solid tumor. Indeed, high retention at the tumor periphery, hindering diffusion to the tumor core, has been demonstrated [204]. Finally, the link between the mNPs and the targeting motifs must be appropriately selected to prevent a loss in their targeted efficacy due to a potential disruption in the recognition moiety.

## 4. Reflections on the Use of mNP for Targeting Tumors

In 2016, Prof. Chan and his team conducted a meta-analysis on targeting (without specifically distinguishing between tumor or cell targeting but focusing on in vivo models), considering 180,000 studies published between 2005 and 2015 [205]. The first noteworthy piece of information (and quite surprising) was that, due to errors in controls and low-quality data, only 117 of them could be included in the final analysis. The second relevant conclusion, with a greater impact on the targeting itself, was that the mean accumulation in the tumor tissue, related to the injected dose, was only 0.7% (irrespective of passive or active targeting). Two years later, the same group quantified the number of mNPs (injected via the tail vein) that interacted with the cancer cells within the tumor mass, determining the astonishing value of 14 out of every 1 × 10^6^ injected, that is, 0.0014% related to the injected dose [206]. Remarkably, this result implies that 99.9986% of the injected mNPs never physically contacted cancer cells. Therefore, although the number of NPs reaching the solid tumor is higher, most of them remain in the ECM or interact with TAMs. To the best of our knowledge, this situation has not improved at all. It is striking that 60 years have passed since the first flight of the Wright brothers to the moon landing of Neil Armstrong, whereas, in the same period, the accumulation of intravenously administered mNPs within solid tumors remains less than 1%. Perhaps the scientific community should consider a drastic shift from the current “publish or perish” mentality towards fostering more innovative scientific ideas. Nonetheless, we must be optimistic, as a smart design of theranostic mNPs with improved features for biomedical applications could lead to success in fighting cancer, even with less than 1% actually targeting tumors, as reported recently by some of us [207].

## 5. Conclusions

**Figure 5:**
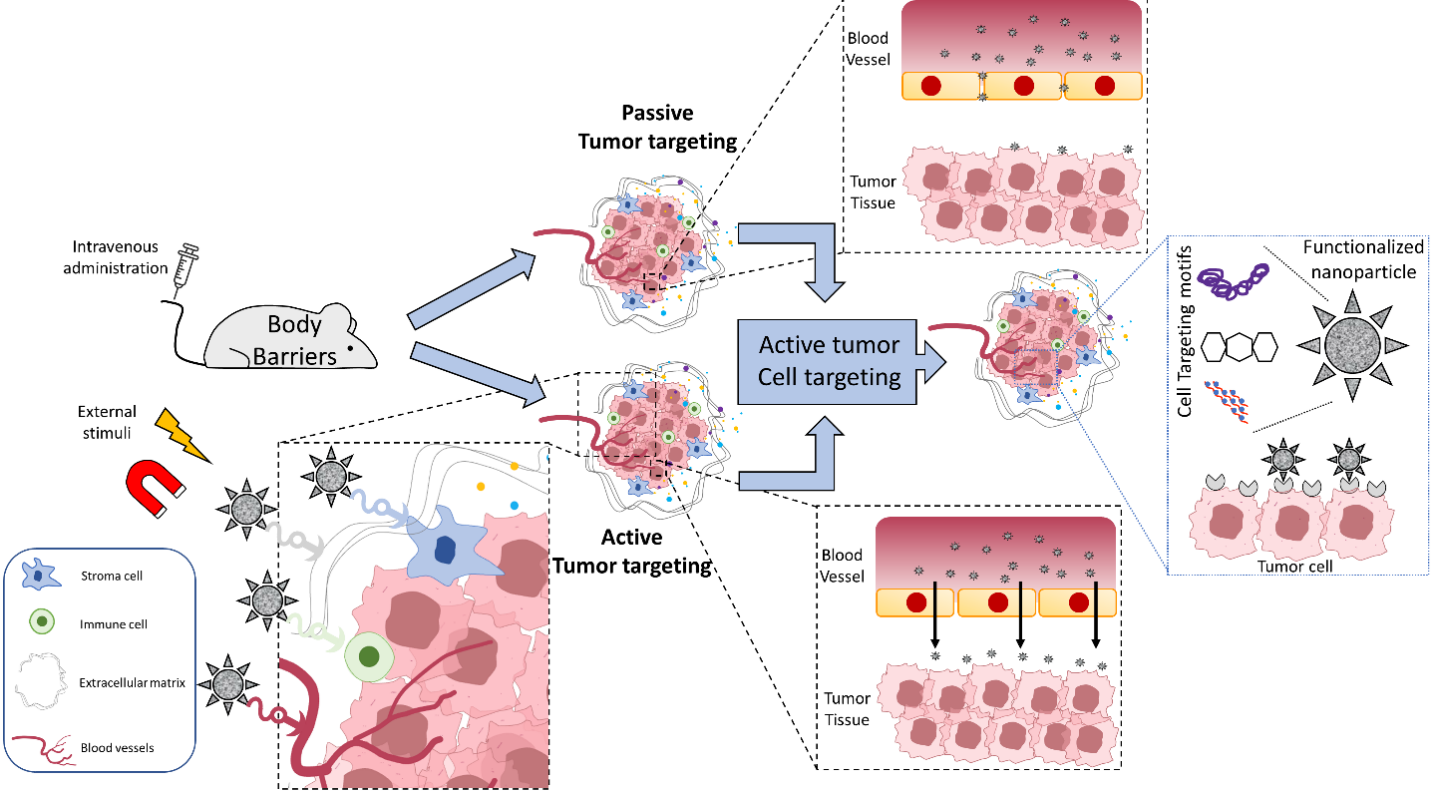
Scheme representing tumor targeting (either passive or active) versus tumor cell targeting.

Nanomedicine applied to oncological patients is a research field with impressive potential, capable of being a turning point in future treatments and, therefore, drastically improving prognosis. However, despite the significant economic effort made in recent decades in this field, the clinical translation of metallic nanoformulations has been extremely limited. To address this issue, it is crucial to perform a smart design from the earliest synthetic steps and to take into account all the problems/barriers that these metallic nanoformulations will face in vivo, especially once they are administered in the bloodstream. To successfully carry out this process, it is essential for researchers to have a clear understanding of all the processes/concepts involved. For example, something as obvious as the difference between tumor targeting and tumor cell targeting in vivo has been blending randomly throughout the literature over the years. In this review, we have endeavored to provide clear definitions of the concepts of passive tumor targeting, active tumor targeting, and active tumor cell targeting (summarized in Figure 5). Furthermore, some key guidelines and considerations that must be taken into account in the design of metal-based nanoformulations to accelerate their translation to the clinical field have been provided and discussed based on our experience. Overall, the following insights can be gleaned from the reviewed literature for the smart design of mNPs for tumor theranostics: the metallic core should have a size below 20–25 nm (small enough to retain the new properties emerging at the nanoscale); the spherical shape seems to perform better, yet further conclusive evidence is needed to establish its preference over other shapes, such as cubical; then a crucial step is the functionalization with molecules that improve their biocompatibility and stealth properties, with PEG being the most commonly used, but the emergence of new coatings capable of improving these aspects is eagerly anticipated. Although negative surface charge is commonly preferred, its role in tumor targeting has not been definitely elucidated. A second functionalization with motifs that provide active tumor targeting capabilities may be required, as it seems to render higher tumor targeting efficacy than passive targeting or other active targeting strategies. Additionally, if the final goal is targeting tumor cells, another functionalization would be needed to specifically recognize molecules overexpressed by tumor cells; and finally, the composition of the core, beyond toxicity concerns, is constrained by the requirement that the resulting mNPs be trackable in vivo by available diagnostic imaging techniques, such as MRI or CT.

## Figures and Tables

**Table 1:** Summary of the tumor targeting studies discussed in this review. ‘N.A.’ indicates information not available. (*) In intratumoral administration, the percentage relative to the injected dose is assumed to be 100%. (**) The authors quantified Fe using ICP, but they did not describe how to differentiate between endogenous and exogenous Fe. (***) Lack of quantification with respect to the injected dose.

mNPs	Size (nm)	Shape	Functionalization	ζ (mV)	HD (nm)	Targeting [% Relative to Initial Dose]	Ref.
IONPs	N.A.	Spherical	hyaluronic acid AND 6-(2- nitroimidazole)hexylamine	N.A.	48 ± 3	Passive [N.A.]	[84]
IONPs	N.A.	Spherical	mPEG-2000-Thioketal(TK)-C16	N.A.	≈27	Passive [>70 µg] **,***	[85]
Fe-Au NPs	19.7 ± 2.8	Quasi-Spherical	3,4-dihydroxyhydrocinnamic acid (DHCA) AND trimethylammonium dopamine (TMAD)	−0.49	≈20	Passive [≈4%]	[86]
IONPs	19.1 ± 1.8	Spherical	PEGylated ligand	N.A.	42.6 ± 1.8	Active (intratumoral) [100%] *	[89]
IONPs	N.A.	Quasi-Spherical	Chitosan AND poly(ε-caprolactone)	≈28	N.A.	Passive AND Active (external stimuli) [N.A.]	[92]
IONPs	≈200	Spherical	Liposomes	N.A.	≈200	Active (external stimuli) [>10 µg] **,***	[93]
AuNPs	≈22	Spherical	2,5-Diphenyltetrazole (Tz) AND methacrylic (Ma) AND polyethylene glycol (PEG)	N.A.	≈35	Active (external stimuli) [≈5%]	[94]
AuNPs	N.A.	N.A.	polyethylene glycol (PEG)	N.A.	N.A.	Active (RGD) [N.A.]	[101]
IONPs	≈10	Spherical	polyethylene glycol (PEG)	N.A.	44.7 ± 0.6	Active (RGD) [N.A.]	[102]
IONPs	≈5	Spherical	generation-5 poly(amidoamine) dendrimers	−5.5	≈530	Active (RGD) [>50 µg] **,***	[103]
IONPs	8.4 ± 0.5	Quasi-Spherical	citric acid	−7.3 ± 0.2	50.1 ± 6.3	Active (Transferrin) [N.A.]	[104]
MSN	≈60	Spherical	polyethylene glycol (PEG) AND tetrazines	≈−10	≈66	Active (Other) [N.A.]	[110]
AuNPs	13.4 ± 1.3	Spherical	polyethylene glycol (PEG)	N.A.	≈25	Active (Other) [4%]	[111]
AuNPs	15 < 100	Spherical	polyethylene glycol (PEG)	N.A.	56 < 154	Active (effect on physiological barriers) [5% < 15%]	[113]
Cu-AuNPs	1.8 ± 0.2	Spherical	polyethylene glycol (PEG)	−15 < 10.8	≈8	Active (effect on physiological barriers) [<2%]	[116]
AuNPs	3 < 120	Spherical	polyethylene glycol (PEG)	−9 < −6	10 < 120	Active (effect on physiological barriers) [<0.5%]	[117]
